# Making every death count: institutional mortality accuracy at Ola During Children’s Hospital, Sierra Leone

**DOI:** 10.11604/pamj.2020.37.356.23607

**Published:** 2020-12-18

**Authors:** Hany Ragab, Andrew Mclellan, Nellie Bell, Ayeshatu Mustapha

**Affiliations:** 1 Paediatrics, Global Links Program, the Royal College of Paediatrics and Child Health, London, United Kingdom,; 2 College of Medicine and Allied Health Sciences, Faculty of Nursing, University of Sierra Leone, Freetown, Sierra Leone,; 3 Faculty of Paediatrics, Ola During Children's Hospital, Freetown, Sierra Leone,; 4 Medical Superintendence, Ola During Children's Hospital, Freetown, Sierra Leone

**Keywords:** Mortality, cause-of-death, epidemiology, paediatrics, children, accuracy, quality, Freetown, Sierra Leone

## Abstract

**Introduction:**

health care data accuracy feeds the development of sound healthcare policy and the prioritisation of interventions in scarce resource environments. We designed a retrospective study at the sole paediatric government hospital in Sierra Leone to examine mortality statistics, specifically: the accuracy of mortality data collected in 2017; and the quality of cause of death (CoD) reporting for 2017.

**Methods:**

the retrospective audit included all available mortality statistics collected at the hospital during the 2017 calendar year. For the purpose of calculating a mortality rate, admission data was additionally gathered. Four different hospital entities were identified that collected mortality data (the Monitoring and Evaluation (M&E) office; the nurse ledgers; the office of births and deaths; and the mortuary). Data from each hospital entity were used for the comparative analysis.

**Results:**

striking differences were found in the rate of hospital mortality reported by different entities. The M&E office (responsible for providing data to the ministry of health and sanitation) reported a hospital mortality rate of 2.94% in 2017. Mortuary and nursing admissions records showed a hospital mortality rate of 18.7%. Discrepancies and issues of quality in CoD reporting between hospital entities were identified.

**Conclusion:**

significant variations were found in the generation of official hospital mortality data. Mortality data informs health service prioritisation, resource distribution, outcome measures and epidemiological surveillance. Resources to support quality improvement initiatives are needed in the creation of an in-hospital system that reports accurate data with a process for real-time institutional data feedback.

## Introduction

Paediatric mortality statistics are the leading data sources used to orchestrate child mortality reduction policies as well as general disease surveillance [1]. Health systems worldwide depend on reliable information regarding mortality data to be able to respond effectively to changing epidemiological circumstances and evaluate the comparative efficacy of different interventions [2]. In the current climate of the COVID-19 global pandemic, now more than ever, reliable mortality data is imperative. Mortality and cause of death (CoD) statistics of poor quality can falsely reassure, distract or mislead policy debates [2].

The “gold standard” in mortality statistics is to have all deaths in health facilities registered, medically certified and coded using the International Classification of Disease (ICD), with an overarching goal of developing a fully functioning Civil Registry and Vital Statistics (CRVS) system [2-4]. The benefit of such a system is that mortality statistics are collected and analyzed continuously (real time, daily in a functioning system) and can be disaggregated by age, sex, CoD and geography [3,5]. Such a system relies on in-hospital CoD reporting determined by physicians or other health workers. This gold standard of certification and coding is both labor and resource intensive and has posed challenges in numerous developing countries [6-12]. Efforts by international actors to empower countries to register every birth and death and to certify every cause of death have been described by some as the single most critical failure of development over the past 30 years [11].

Data about deaths in health facilities may not be nationally representative at the population level. Nonetheless, the medical certification and coding of deaths and compilation of facility mortality statistics provide valuable benefits, including improved quality of care and the availability of continuous real time data to inform national health planning and policy [2,13,14]. Reliable data on facility-based care and mortality outcomes, including CoD, supports the development of an integrated intelligence-led health system and enables appropriate resource distribution within the system. Without trustworthy baseline data innovative healthcare research cannot flourish and the efficacy of interventions may not be accurately interpreted; indeed, potentially successful interventions may even be missed or discontinued [2].

**Our study:** working at a paediatric hospital in Freetown, Sierra Leone as clinical staff (from: the Royal College of Paediatric and Child Health Global Links Programme; The University of Sierra Leone´s College of Medicine and Allied Health Sciences; and senior hospital faculty), we noted that the mortality data reported to the ministry of health and sanitation from the hospital did not appear to reflect the “on the ground” clinician experience. The hypothesis was that the official mortality data gathering measures were quantitatively inaccurate. Additionally, we were curious regarding the more qualitative aspects of CoD reporting. We designed a retrospective study to examine the hospitals mortality statistics, specifically: the accuracy of mortality data collected in 2017; and CoD reporting for 2017.

**Study setting:** the study was carried out at Ola During Children's Hospital (ODCH); Sierra Leone´s sole paediatric referral centre. The government teaching hospital has 200 beds and is situated in the east end of the capital, Freetown. The hospital has received partial accreditation by the West African College of Physicians for postgraduate training in paediatrics. The hospital inpatient department includes two general wards as well as a Special Care Baby Unit (SCBU), a therapeutic feeding unit, a paediatric intensive care unit, a high dependency unit and an emergency department.

**Ethics approval:** the study protocol was imbedded in a larger project regarding emergency triage assessment and treatment directed by the Royal College of Paediatrics and Child Health that received ethics approval from the Sierra Leone Ethics and Scientific Review Committee.

## Methods

A retrospective audit of all available mortality statistics collected at the hospital during the 2017 calendar year was undertaken. The study began with identifying the systematic ways in which death is reported within the hospital. Interviews were conducted with each hospital department to discover if and how they collected mortality data. This allowed the identification of 4 different in-hospital entities that collect mortality data. Below is a description of how each entity collects and reports mortality statistics: the Monitoring and Evaluation (M&E) department collects mortality data through its departmental officers who upload the data to a national database. The day to day staffing of the service relies on one member of staff in the medical records department and one member of staff in the M&E department. This data is provided to the ministry of health and sanitation where it is then incorporated in the national calculation of mortality. The department collects the data through ministerial registers placed in each hospital ward. The information regarding each admission to a ward is recorded on the ministerial register by the nursing staff while on shift at the time of the admission. The data are collated in handwritten summaries by the M&E officer prior to electronic upload; the nursing superintendents collate mortality data from the nursing ledgers from each hospital department and ward and record numbers daily in a statistics folder; the office of births and deaths situated on site for the purpose of the Children´s and co-located Maternity Hospital collects original death certificates from parents/guardians and provides in exchange, a right to burial form.

The office of births and deaths specifically serves the hospitals. Its office hours are Monday to Friday 9am to 5pm. A record of death certificates is available at the office and the right to burial form is theoretically needed to collect the body from the mortuary; the hospital mortuary situated on site serves the Children´s Hospital and co-located Maternity Hospital. There were no community cases transferred to this mortuary during the period of study. The mortuary records data in a ledger that identifies: the name and age of the child brought to or collected by the mortuary staff; the hospital ward the body came from; the date of arrival of the body; and the name of the destination cemetery. Mortality records for 2017 were collected from each of the in-hospital entities for analysis. For the purpose of calculating a mortality rate, admission data were gathered from the ledger of daily statistics at the nursing superintendent’s office and from the M&E records.

**Analysis:** data captured from each hospital entity recording mortality were anonymized and stored in Microsoft Excel files. Comparative analysis of quantitative crude data from each mortality data source was completed and involved calculating simple percentages, proportions and constructing charts. Additional analysis of variables such as age, ward and day of week were conducted. Using Microsoft Excel and the analysis toolpack add-in, descriptive statistics, Chi squared and single factor ANOVA tests were conducted on appropriate data subsets. Qualitative data regarding CoD reporting are presented descriptively.

## Results

**Figure 1:**
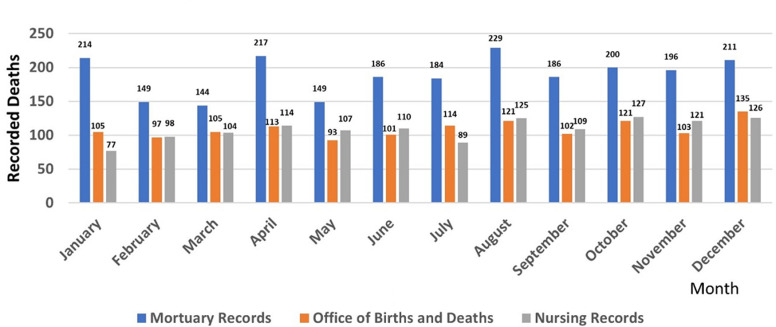
crude mortality per month by differing hospital data sources, of note “monitoring and evaluation” data not represented; 408 deaths per annum with average of 34 deaths per month

**Mortality by data source:** the M&E office end of year summary concluded there were 408 deaths which equates to an average of 34 deaths per month. Monthly reports were not available. The office of births and deaths have 1306 death certificates collected over the course of 2017. The nursing department recorded 1309 deaths in 2017. The mortuary recorded 2266 in hospital deaths in 2017. Monthly reports from these three data sources were available (Figure 1). From this we found that the hospital mortality being reported by the department (M&E) officially designated to report to the national database appears to be capturing less than 20% of the actual mortality in 2017. The difference in mortality reporting between in-hospital entities were significant at p<0.001 on Chi squared analysis.

**Figure 2:**
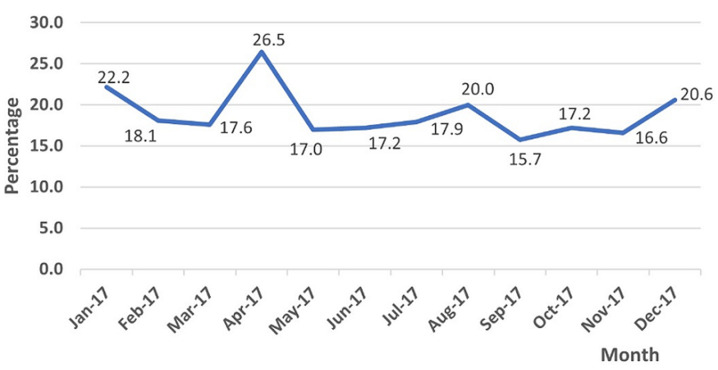
monthly mortality rate at Ola During Children's Hospital in 2017 using mortuary recorded deaths as a percentage of total monthly admissions to the hospital

Admission data were selected as the denominator for the generation of a mortality rate. The M&E department recorded 13,855 diagnostic inpatient cases in 2017. The nursing records from the emergency admissions portals and the superintendent´s office recorded 12,114 admissions in the same year. The annual mortality rate as a proportion of admissions according to the M&E office was 2.94%. Using mortuary data and nursing admissions data we generated an in-hospital paediatric mortality rate of 18.7% for the year. Mortuary monthly crude data and nursing records of monthly admissions show a monthly mortality rate range between 15 and 26% of admissions (Figure 2). For the purposes of subgroup analysis, we adopted mortuary data and nursing admission data as reference values.

**Figure 3:**
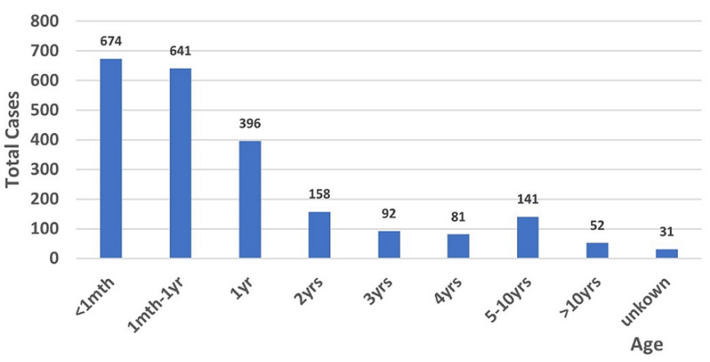
2017 crude mortality by age group as registered by Ola During Children’s Hospital Mortuary

**Mortality descriptive analysis:** mortality was analyzed by age distribution (Figure 3), hospital ward and day of the week from the mortuary ledger. Day of the week data showed a significant difference in mortality distribution with highest crude mortality occurring on Thursday, Friday and Saturday (Chi-square p=0.04). An analysis of crude mortality by hospital ward found a significant neonatal and special care baby unit (SBCU) contribution to overall mortality burden (p<0.001, Chi-square) with 2417 admissions to SCBU and a unit mortality rate of 25.5%.

**Cause of death analysis:** with regards to the CoD analysis, two data sources were found to collect the information, the M&E department and the office of births and deaths via death certificates. The M&E department use the ward ministerial registers as their primary source of information. The patient´s information on these registers is completed by nursing staff on hospital admission using the emergency department´s working diagnosis. The database and diagnostic fields used nationally are designed for an adult population. The five most common causes of death according to M&E data in 2017 are; other (215), malaria (99), respiratory infection (47), malnutrition (25), anaemia (11).

**Figure 4:**
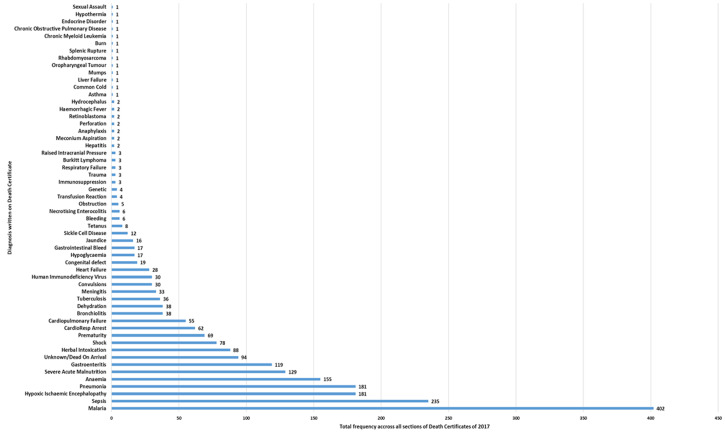
death certification diagnostic frequency for cause of death at Ola During Children's Hospital in 2017

According to death certification data in 2017 the five most common causes of death by frequency across all sections of the certificate were; malaria (402), sepsis (235), hypoxic ischaemic encephalopathy (181), pneumonia (181) and anaemia (155). Table 1 summarizes the quantitative differences in CoD reports found (using the M&E national database of diagnostic categories) between the M&E department and official death certificates. Single factor ANOVA analysis showed these differences to be significant at p<0.005. Table 1 also includes the highest frequency diagnoses present on death certification (denoted with double asterisk) that are not represented in the M&E national database of diagnostic categories. The CoD data from death certification records are summarized in Figure 4.

**Table 1:** comparative cause of death data for 2017 at Ola During Children's Hospital by “monitoring and evaluation” national database diagnostic categories

	Monitoring and evaluation department	Cause of death (death certification)
Acute abdomen	0	7
Anaemia	11	155
Appendicitis	0	0
Acute respiratory infection	47	181
Burns	0	1
**Bronchiolitis	Not recorded	38
**Congenital disease	Not recorded	23
Cardiovascular disease	0	28
Cholera	0	0
Diabetes	0	0
Diarrhoea	5	119
**Gastrointestinal bleed	Not recorded	17
Hepatitis	1	2
**Herbal intoxication	Not recorded	88
**Hypoxic ischaemic encephalopathy	Not recorded	181
Human immunodeficiency virus	0	33
**Hypoglycaemia	Not recorded	17
**Jaundice	Not recorded	16
Malaria	99	402
Severe acute malnutrition	25	129
Meningitis	3	33
**Prematurity	Not recorded	69
**Sepsis	Not recorded	235
Tuberculosis/leprosy	2	36
Tetanus	0	8
Trauma	0	5
Tumour/cancer	0	8

## Discussion

Health care data accuracy feeds the development of sound healthcare policy and the prioritisation of interventions in all healthcare systems but is acutely important in scarce resources environments. The study focused on the very grass roots of data collection at one institution. The study results may highlight some shared in-country challenges in the accuracy of mortality data collection and reporting that public health policy makers may fail to appreciate in smaller hospitals, and rural and community settings.

**Quantitative accuracy:** the study´s findings confirm other research outcomes that indicate that in many developing countries, mortality statistics systems are weak, often characterized by fragmented and uncoordinated collections from multiple sources [3]. As a result, many developing countries lack continuous, permanent and universal sources of mortality data and thereby face considerable challenges in: building responsive health systems; measuring and monitoring mortality; and reporting against national and global development mortality goals and targets, such as those set by the sustainable development goals.

We found significant (p<0.001, ANOVA analysis) intra-data source variation in numbers recorded for in-hospital mortality. The mortuary had names, ages and origins of 2266 deaths, with only around half of these captured by the nursing department and office of births and deaths and less than a quarter of which were captured by official data recording tools. We could not identify coordinated efforts of quality assurance such as the triangulation or cross checking of findings between in-hospital entities. As is often the case in large complex institutions, sub-entities within that institution have their own individual and wholly separate functional rationale, mode of working and reasoning when it comes to information capture, accuracy and quality assurance. Each entity had its own specific challenges with regards to case ascertainment: the M&E department relies on ministerial registers that are completed by nursing staff for each admission to their unit. This can be prone to missing some data capture in a low staffing/high acuity environment.

The diagnostic columns in the register are completed at the time of admission and often report the working diagnosis of the house officer (junior physician in training) from the emergency department. The diagnostic column is not later updated; the nursing department relies on nursing ledgers containing patient details by bed and by ward which is reported daily. This daily recording is a snapshot of the caseload in that department at the time of recording in the ledger and can miss high turnover activity during the day and night and the task itself can be missed in periods of low staffing/high acuity. There is a potential point of contact here in data sources in that the same nurse may have to both record information in the ministerial register and update the nursing ledger; the office of births and deaths has limited working hours and as such is not always available for families to visit following a death; the mortuary relies on a physical body transferred into their department, however, even this can have limitations, as it was reported by hospital staff that families at times have removed the body directly from wards, wrapped in sheets and transferred home or to a burial site.

**Qualitative accuracy:** the CoD data shows the largest mortality burden falls in the neonatal period, however, neonatal specific categories are not represented in the adult-centric national database of mortality. As neonatal outcomes become, proportionally, more important in national and international paediatric survival strategies, this data and knowledge gap will become increasingly problematic. Death certificates show the majority of mortality burden is not being adequately described by the current database categories, with significant contributors such as sepsis, prematurity, hypoxic ischemic encephalopathy and herbal intoxication lacking representation and likely being reported in the “other” category.

Qualitative accuracy of death certification, closely allied to that of medical record keeping is a well-known issue in resource limited environments [15-17]. There are significant limiting factors in fulsome CoD reporting at ODCH. Death certificates are largely completed by junior staff, who lack the experience in understanding the probable diagnoses. There are limited diagnostic tools to confirm suspected diagnoses. In cases of an unclear diagnosis prior to death there are few possibilities for postmortems, due to limited access to pathologists and histo-pathologists and cultural/religious requirements regarding the timing of the burial. Furthermore, in a health system lacking in post-discharge responsibilities and longer term follow up, where patients improve and survive to discharge, the rationale for spending scarce time and resources on improved diagnostics may continue to be weak.

When it comes to paediatric mortality it is wise to note still birth data, for which the mortuary had a separate record. We noted that still births in some months equaled neonatal deaths and represent a significant ongoing burden. When examining death certification, we saw several major contributors who are not represented at all by the current national database. Despite the limitations inherent in death certification we found the data from death certificates to prove a richer source of causes of mortality than the M&E data.

**Local and national strategies:** at ODCH, influenced by the study results, a new strategy was developed for data collection using the mortuary records as the “gold standard” resource for triangulation. Morbidity and mortality meetings with weekly data feedback and trend analysis methodologies have been established. This live feedback of institutional data has led to insight allowing for changes in management, for example shift patterns and staffing distributions were altered to cover high risk units and times of the day. Regional and national level dissemination of study results have influenced a discussion and an engagement in developing a paediatric specific national mortality database with categories that could be mapped to ICD10 classifications.

**Limitations:** the study focused on one hospital; it is however, the country´s only tertiary paediatric hospital and its data collection methodology and accuracy may be representative of what is occurring in other health care facilities nationally. Analysis on CoD data relied on M&E database and death certificates which were both found to have flaws. Given the level of missing data, it is difficult to be confident that death certificates provide an accurate snapshot and representative sample of the distribution of causes of death, however, anecdotally based on healthcare worker experience, this data appears more representative of the clinical activity than the M&E database. The examination of clinical case notes would have provided a richer overall analysis. There are however, no national system of health codes or hospital code identifiers for patients, nor a system of national identification numbers, as such, each visit to hospital generates a new hospital identification number and once discharged these notes are stored by code. Without hospital ward clerks and modernization of health care records, ledgers and registries will remain the current primary hospital data source.

## Conclusion

The accuracy of the rate and cause of child mortality is crucial to how a nation provides care and ultimately sustains life. Quality assurance needs to be embedded in each operational stage in the production of mortality statistics [3,15]. Accurate mortality statistics are a primary requirement for the management of national health programs, including defining immediate interventions for disease outbreaks and epidemics. The recent ebola outbreak demonstrates the need for real time mortality data to be in place in all countries. Mortality statistics improvement in Africa is critical to future health development [2,3,11,14]. The sustainable development goals focus attention on the need for accurate overall and cause-specific mortality data [2]. Accuracy is key to highlighting areas for prioritization to achieve the largest impact on childhood survival in Sierra Leone. The importance of mortality data lies in the fact that it informs health service prioritisation, resource distribution, outcome measures and epidemiological surveillance. Further resources and energy are required to create a system that reports quantitatively and qualitatively accurate data with a process for real time institutional data feedback at ODCH. A national dialogue in Sierra Leone has begun on increasing resource allocation to healthcare data collection and revising the adult centric national mortality database to include a paediatric focus. It remains to be seen whether the unification of mortality data collection methodology within healthcare institutions or quality assurance methodologies such as data triangulation strategies can be scaled up from local to national levels. While longer term issues of social security identifiers, electronic medical records and permanent health care patient identifiers are discussed at the highest national level, more short-term interventions of low tech and low cost should be considered in the interim to ensure every death is counted and counts.

### What is known about this topic

- Paediatric mortality statistics are the leading data source used to orchestrate child mortality reduction policies as well as general disease surveillance;
- The “gold standard” in mortality statistics is to have all deaths in health facilities registered, medically certified and coded using the International Classification of Disease (ICD), with an overarching goal of developing a fully functioning Civil Registry and Vital Statistics (CRVS) system;
- In the current climate of the COVID-19 global pandemic, now more than ever, reliable mortality data is imperative.

### What this study adds

- The study´s findings confirm other research outcomes that indicate that in many developing countries, mortality statistics systems are weak, often characterized by fragmented and uncoordinated collections from multiple sources;
- Death certificates show the majority of mortality burden is not being adequately described;
- The need for developing a paediatric specific national mortality database with categories that could be mapped to ICD10 classifications.
